# Effect of surface treatment of prefabricated teeth on shear bond strength of orthodontic brackets

**DOI:** 10.1590/2177-6709.22.4.047-052.oar

**Published:** 2017

**Authors:** Marina Cumerlato, Eduardo Martinelli de Lima, Leandro Berni Osorio, Eduardo Gonçalves Mota, Luciane Macedo de Menezes, Susana Maria Deon Rizzatto

**Affiliations:** 1 Pontifícia Universidade Católica do Rio Grande do Sul, Faculdade de Odontologia (Porto Alegre/RS, Brasil).; 2 Pontifícia Universidade Católica do Rio Grande do Sul, Faculdade de Odontologia, Disciplina de Ortodontia (Porto Alegre/RS, Brasil).; 3 Universidade Federal de Santa Maria, Faculdade de Odontologia, Departamento de Estomatologia (Santa Maria/RS, Brasil).; 4 Pontifícia Universidade Católica do Rio Grande do Sul, Faculdade de Odontologia, Disciplina de Materiais Dentários (Porto Alegre/RS, Brasil).

**Keywords:** Artificial teeth, Orthodontic brackets, Shear strength, Temporary crowns.

## Abstract

**Objective:**

The aim of this *in vitro* study was to evaluate and compare the effects of grinding, drilling, sandblasting, and ageing prefabricated teeth (PfT) on the shear bond strength (SBS) of orthodontic brackets, as well as the effects of surface treatments on the adhesive remnant index (ARI).

**Methods:**

One-hundred-ninety-two PfT were divided into four groups (n = 48): Group 1, no surface treatment was done; Group 2, grinding was performed with a cylindrical diamond bur; Group 3, two drillings were done with a spherical diamond bur; Group 4, sandblasting was performed with 50-µm aluminum oxide. Before the experiment, half of the samples stayed immersed in distilled water at 37^o^C for 90 days. Brackets were bonded with Transbond XT and shear strength tests were carried out using a universal testing machine. SBS were compared by surface treatment and by ageing with two-way ANOVA, followed by Tukey’s test. ARI scores were compared between surface treatments with Kruskal-Wallis test followed by Dunn’s test.

**Results:**

Surface treatments on PfT enhanced SBS of brackets (*p*< 0.01), result not observed with ageing (*p*= 0.45). Groups II, III, and IV showed higher SBS and greater ARI than the Group 1 (*p*< 0.05). SBS was greater in the groups 3 and 4 (drilling, sandblasting) than in the Group 2 (grinding) (*p*< 0.05). SBS and ARI showed a positive correlation (Spearman’s R^2^= 0.57; *p*< 0.05).

**Conclusion:**

Surface treatment on PfT enhanced SBS of brackets, however ageing did not show any relevance. Sandblasting and drilling showed greater SBS than grinding. There was a positive correlation between SBS and ARI.

## RESUMO

**Objetivo:**

o objetivo desse estudo *in vitro* foi avaliar e comparar os efeitos de desgastes, perfurações, jateamento e envelhecimento de dentes pré-fabricados (Dpf) na resistência ao cisalhamento (RC) de braquetes ortodônticos, bem como os efeitos dos tratamentos de superfície no índice de adesivo remanescente (ARI).

**Métodos:**

192 Dpf foram divididos em quatro grupos (n = 48): Grupo 1, sem tratamento; Grupo 2, desgaste de sulcos com broca diamantada cilíndrica; Grupo 3, duas perfurações com broca diamantada esférica; e Grupo 4, jateamento com partículas de óxido de alumínio de 50µm. Antes do experimento, metade das amostras ficou imersa em água destilada a 37^o^C durante 90 dias. Os braquetes foram colados com Transbond XT e os testes de cisalhamento foram realizados com uma máquina universal de ensaios. A RC foi comparada, por tratamento de superfície e por envelhecimento, utilizando-se análise de variância e teste de Tukey. Os escores ARI foram comparados entre os tratamentos de superfície usando o teste Kruskal-Wallis e o teste de Dunn.

**Resultados:**

os tratamentos de superfície aumentaram a RC dos braquetes (*p* < 0,01) e o envelhecimento não aumentou a RC (*p* = 0,45). Os Grupos 2, 3 e 4 mostraram maior RC e maior ARI do que o Grupo 1 (*p* < 0,05). A RC foi maior nos Grupos 3 e 4 (perfuração, jateamento) do que no Grupo 2 (desgaste) (*p* < 0,05). RC e ARI apresentaram correlação positiva (R^2^ = 0,57; *p* < 0,05).

**Conclusão:**

o tratamento de superfície dos Dpf aumentou a RC dos braquetes; porém, o envelhecimento não foi relevante. O jateamento e a perfuração mostraram maior RC do que o desgaste. Existe uma correlação positiva entre RC e ARI.

## INTRODUCTION

In comprehensive orthodontic treatments associated with prosthetic rehabilitation, the orthodontists need to bond brackets on temporary crowns, as the definitive restorations are usually placed after the orthodontic movement.^1^ ^,^ ^2^ Likewise, in management of dental spacing related to extractions, traumatic loss or missing teeth, the brackets are bonded to esthetic pontic materials, in order to provide adequate esthetics during orthodontic treatment.^3^

Prefabricated teeth (PfT) may be customized as temporary crowns or pontics and receive bonded orthodontic brackets. However, shear bond strength (SBS) must withstand the orthodontic forces and masticatory stress.^1^ Bond failures increase the chair time and the costs, compromising the efficiency and effectiveness of the orthodontic treatment, besides being inconvenient for patients.^3^ ^,^ ^4^

Green stones, sandpaper discs, silica carbide paper, sandblasting, laser irradiation, and burs have been used to increase SBS of orthodontic brackets.^1^ ^-^ ^3^ ^,^ ^5^ ^-^ ^10^ In studies carried out with PfT, the use of sandblasting resulted on SBS of 5.5 MPa,^3^ while a extensive wear on the buccal surfaces increased SBS up to 17 MPa.^8^ Orthodontists would rather use a simple method to reach SBS compatible with the clinical needs, avoiding inconvenience for the patients and delay in the treatment. 

From this standpoint the aim of this *in vitro* study was to evaluate and compare the effects of grinding, drilling, sandblasting, and ageing PfT on SBS of orthodontic brackets, as well as the effects of surface treatments on the adhesive remnant index (ARI). The null hypothesis was that there is no difference in SBS and ARI regarding different surface treatments or between aged and non-aged PfT.

## MATERIAL AND METHODS

The sample size calculation indicated 24 specimens in each group to detect a difference of 0.66 MPa (4.22 ± 1.15 MPa)^8^ with a power of 80%, and bilateral alpha level of 5% (Statistical Solutions, LLC Systems, Cottage Grove, WI, USA). One-hundred-ninety-two prefabricated upper central incisors of polymethylmethacrylate (PMMA) with interpolating polymer network (IPN) (Biotone IPN, Dentsply, Petrópolis/RJ, Brazil) were randomly divided into four groups (n = 48), according to the performed surface treatment (Table 1). Samples were submitted to two storages process: immediately bonding (no ageing) and ageing before bonding procedures.

**Table 1:** Groups formed according to the surface treatment of prefabricated teeth (PfT).

Group	Surface treatment performed	n
1	No treatment	48
2	Grinding parallel grooves, with a cylindrical diamond bur	48
3	Drilling two cavities, with a spherical diamond bur	48
4	Sandblasting, with 50-µm aluminum oxide	48

The PfT were mounted into acrylic resin blocks, prepared inside PVC tubes of 20 mm in height and diameter. Samples for ageing groups underwent to a 90-day ageing period, immersed in distilled water at 37°C, in a culture incubator (Fanen, São Paulo/SP, Brazil).^11,12^ Specimens in the Group 1 (control) had no surface treatment. In the Group 2, four parallel grooves were performed on the buccal surfaces, from the left to the right, with a cylindrical diamond bur (2143, KG Sorensen, Cotia/SP, Brazil) mounted in a high-speed handpiece (Kavo, Joinville/SC, Brazil) without water-cooling. In the Group 3, two drillings were done at the center of the buccal surface, until to the depth of the bur tip, with a spherical diamond bur (1012, KG Sorensen) mounted in a high-speed handpiece (Kavo) without water-cooling. In the Group 4, a sandblaster (Microjato, BioArt, São Carlos/SP, Brazil) was used to apply 50-µm aluminum oxide for 10 seconds, at a perpendicular distance of 1 cm from the buccal surface of the PfT.

The bonding procedure followed the same sequence in all groups. The buccal surfaces were etched using 37% phosphoric acid (Villevie, Joinville/SC, Brazil) for 20 seconds, rinsed with distilled water and dried with air-jet. Metal brackets (10.30.201, Morelli, São Paulo, SP, Brazil) were bonded with Transbond XT adhesive (3M Unitek, Monrovia, CA, USA), according to the manufacturer instructions. Brackets were pressured (300 g) with the aid of a customized device, in order to standardize the thickness of the adhesive.^13^ Excess adhesive was removed using a dental probe. The resin adhesive was light-polymerized for 15 seconds in each side of the bracket, with a conventional LED-curing unit (RaddiCal, SDI, Bayswater, Victoria, Australia). Thereafter, the samples stayed immersed in distilled water at 37^o^C, during a 7-day period. 

SBS test was performed using a universal testing machine (EMIC 2000, São José dos Pinhais/PR, Brazil) with a crosshead speed of 0.5 mm/min. The force was applied between the wings and base of the brackets and ran parallel to the PfT buccal surfaces, in occlusal-cervical direction. SBS (MPa) was maximum shear force (N) divided by the bracket base area (14.82 mm^2^).

After debonding, samples were observed with a four-fold magnifying glass, in order to assess the adhesive remnant index (ARI): 0 = no adhesive left on the tooth; 1 = less than half of the adhesive left on the tooth; 2 = more than half of the adhesive left on the tooth; and 3 = all of the adhesive left on the tooth. 

### Statistical analysis

Data were analyzed with SPSS statistical software (version 20.0, IBM, Armonk, NY, USA). Kolmogorov-Smirnov and Shapiro-Wilk tests revealed that data were not normally distributed, requiring logarithmic transformation of the dependent variable (SBS).^14^ The normalized data of SBS were compared between groups (ageing and surface treatment) using two-way ANOVA and multiple comparisons Tukey’s test. The ARI scores were compared between groups (surface treatment) using nonparametric Kruskal-Wallis test and Dunn multiples comparison in order to find out the homogeneous subsets. Spearman’s correlation was employed to verify if the SBS was correlated with ARI scores. Results were significant at the 95% confidence level.

## RESULTS

Table 2 shows that surface treatment on PfT enhanced SBS of orthodontic brackets (*p*< 0.01), unlike the ageing process (*p*= 0.45). Table 3 reveals that surface treatments by drilling (Group 3) or sandblasting (Group 4) showed higher SBS than the grinding (Group 2) (*p*< 0.05). Under the same surface treatment, there was no significant difference between aged and non-aged samples (*p*> 0.05) (Table 4). However, the interaction between factors was statistically significant, revealing that some influence occurred on the process (*p*< 0.05) (Table 2).

**Table 2:** Two-way ANOVA on transformed data.

Dependent variable Shear bond strength	Type III Sum of Squares	df	Mean Square	F	Significance
Corrected model	23.52 ^A^	7	3.36	39.5	< 0.01
Intercept	62.46	1	62.46	734.47	< 0.01
Ageing	0.049	1	0.04	0.57	0.45
Surface treatment	22.58	3	7.52	88.49	< 0.01
Interaction	0.89	3	0.29	3.5	0.017
Error	15.64	184	0.08		
Total	101.63	192			
Corrected total	39.17	191			

A R^2^= 0.6 (Adjusted R^2^= 0.585)

**Table 3:** Descriptive statistics of shear bond strength (SBS), interaction among groups.

			Shear Bond Strength		
		n	Lower limit (MPa)	Upper limit (MPa)	Mean ± SD (MPa)
Factor					
No ageing	-	96	4.51	6.13	5.32 ± 3.98 ^NS^
Ageing	-	96	4.83	6.73	5.78 ± 4.68 ^NS^
Surface treatment	Group				
No treatment	1	48	1.1	1.69	1.39 ± 1.02 ^A^
Grinding	2	48	3.78	5.68	4.72 ± 3.29 ^B^
Drilling	3	48	6.26	7.99	7.12 ± 2.97 ^C^
Sandblasting	4	48	7.56	10.35	8.59 ± 4.81 ^C^

Multiple comparisons Tukey’s test performed on treatment groups. Different letters indicate statistical difference (p < 0.05).

**Table 4:** Descriptive statistics of shear bond strength (SBS).

Surface treatment	Group	Ageing	Mean ± SD (MPa)	Significance
No treatment	1	No	1.25 ± 0.98	NS
		Yes	1.55 ± 1.06	
Grinding	2	No	5.56 ± 3.78	NS
		Yes	3.90 ± 2.55	
Drilling	3	No	6.79 ± 3.51	NS
		Yes	7.46 ± 2.33	
Sandblasting	4	No	7.70 ± 3.51	NS
		Yes	10.21 ± 5.63	

NS = non-significant statistical difference (*p* > 0.05); SD = standard-deviation; MPa = megapascal.

Table 5 shows that ARI scores were statistically greater in the Groups 2, 3 and 4 (treated surface) than in the Group 1 (no surface treatment) (*p*< 0.01). All samples in the Group 1 showed ARI = 0. In the Group 3, 96% of the sample showed ARI = 1. In the Groups 2 and 4, there was a wider variation in ARI scores. In the Group 2, ARI = 0 occurred in 52% of the sample, ARI = 1 in 27%, and ARI = 2 in 21%. In the Group 4, ARI = 0 was observed in 33% of the sample and ARI = 2 or 3 in 44%. A positive correlation was observed between SBS and ARI scores (Spearman’s correlation R^2^= 0.571; *p*< 0.05).

**Table 5:** ARI scores.

		ARI				
Group	n	0	1	2	3	Significance
1 - No treatment	48	48	0	0	0	A
2 - Grinding	48	25	13	10	0	B C
3 - Drilling	48	0	46	2	0	D
4 - Sandblasting	48	16	11	18	3	C D

Kruskal-Wallis, difference among groups was statistically significant (*p* < 0.001). Dunn multiples comparison identified the homogeneous subsets. Different letters indicate statistical difference.

## DISCUSSION

SBS of orthodontic brackets bonded to PfT was affected by the mechanical surface treatments but not by the ageing process used in this *in vitro* study. The null hypothesis was partially rejected, because a statistical significant difference was found among surface treatments but not regarding ageing. Analysis of correlation indicated that as SBS increased, ARI scores were higher. 

PfT was used to test SBS of orthodontic brackets,^3^ ^,^ ^8^ instead of samples of bis-acryl, polymethylmethacrylate, and methacrylate resins.^1^ ^,^ ^5^ ^-^ ^7^ ^,^ ^9^ ^,^ ^10^ Hand-made pontics are time consuming and require unusual materials to the orthodontists.^3^ On the other hand, the PfT are offered in a variety of shapes, sizes and colors, being easily customized to provide adequate esthetics during the orthodontic treatment. The surface treatments of grinding, drilling and sandblasting of PfT were useful in increasing SBS of orthodontic brackets. However, SBS should reach 6 to 8 MPa to withstand the orthodontic and masticatory forces.^1^ ^-^ ^3^ ^,^ ^15^ Weak SBS values observed in the Group 1 (untreated surfaces) probably occurred due to a high density of the PfT and the different chemical structure of the resin adhesive used (bis-GMA).^16^ In the present study, sandblasting and drilling increased SBS until the clinical needs. On the other hand, grinding resulted in low SBS values. Perhaps, a more invasive grinding procedure is required.^8^

**Figure 1:**
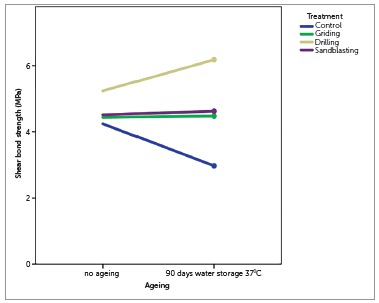
Behavior of SBS means of the groups.

In the present study, the 90-day ageing period caused increase on SBS of orthodontic brackets, however without statistical significance. Chay, Wong, Mohamed et al.^1^ reported an increase in SBS after one week of PMMA ageing. Immersed in water, PfT undergo an imbibition that progressively separates the polymer chains, decreasing its hardness.^17^ In the present study, it was observed that ageing of samples decreased SBS in the Group 1 and increased the SBS in the Group 3 (Fig 1). This variation could justify the statistical significance of interaction observed in the ANOVA (Table 2).

The ARI scores were zero for all specimens of the Group 1 (no treatment), indicating that no composite resin remained on the PfT surfaces. This result is in line with the findings of Blakey and Mah.^2^ Sandblasting and drilling showed no statistical significant difference in ARI scores, being in accordance with the results for SBS. In a similar way, the sandblasting and grinding groups showed a wider variation in ARI and greater standard deviations in SBS (Tables 3 and 5). Although SBS for grinding (Group 2) was statistically different from the other groups, a positive statistically significant correlation was found (R^2^= 0.571) between ARI assessments and SBS values, what is in agreement with other studies.^18^ ^,^ ^19^

Based on results of this study, one could elect sandblasting or drilling of PfT to increase SBS of orthodontic brackets. However, applying intraoral sandblasting in cemented temporary crowns might be harmful for the patient, due to the particles of aluminum oxide suspended in the air. In these cases, the drilling with a spherical bur is wiser choice. When a pontic is replaced to renew the esthetics during the orthodontic treatment, drilling would be a simpler method to increase SBS, eliminating the need of a sandblaster at the office.

The complete reproduction of the oral environment is not possible, but *in vitro* studies are useful to determine the strength of a given bonding technique, with acceptance of some limitations.^4^ In the present study, only mechanical surface treatments were tested, despite the chemical bond possibility using acrylic resin.^10^ However, the *in vivo* manipulation of acrylic monomer might be cytotoxic, acting as an adjuvant agent at either the sensitization or elicitation step in the allergic dermatitis induced by nickel.^20^ ^-^ ^22^ Ageing process was performed with water instead of saliva, once water is the principal compound of the saliva. Moreover, water easily penetrate the polymer and provoke hydrolysis in the PfT polymeric chains.^23^

Future studies could investigate if a retentive cavity drilled with an inverted cone bur is even more effective in increasing orthodontic SBS.^7^ The present study outcomes demand a cautious extrapolation to clinical situations, as *in vitro* studies fail to reproduce the oral environment. However, *in vitro* studies are crucial to indicate which hypothesis would be best suited in a clinical trial.^24^ ^,^ ^25^

## CONCLUSIONS

» The null hypothesis was partially rejected.
» The surface treatments of PfT increased SBS of brackets, unlike the ageing process.
» Drilling and sandblasting showed a greater increase in SBS than the grinding.
» The surface treatments increased the ARI scores.
